# A Machine Learning Approach to Build and Evaluate a Molecular Prognostic Model for Endometrial Cancer Based on Tumour Microenvironment

**DOI:** 10.1111/jcmm.70316

**Published:** 2025-02-21

**Authors:** Di Wu, Zhifeng Yan, Mingxia Li, Mingyang Wang, Yuanguang Meng

**Affiliations:** ^1^ School of Medicine Nankai University Tianjin China; ^2^ Department of Obstetrics and Gynecology The First Affiliated Center of Chinese People's Liberation Army (PLA) General Hospital Beijing China; ^3^ Department of Obstetrics and Gynecology The Seventh Medical Center of Chinese PLA General Hospital Beijing China

**Keywords:** endometrial carcinoma, immunohistochemistry, machine learning, molecular, prognosis, tumour microenvironment

## Abstract

Endometrial cancer (EC) incidence and the associated tumour burden have increased globally. To build a molecular expression prognostic model based on the tumour microenvironment to guide personalised treatment using a machine learning approach. Two datasets were reviewed, including a training cohort (*n* = 698) and a testing cohort (*n* = 151). All patients underwent hysterectomy ± adnexectomy ± lymph nodes dissection between December 2014 and June 2020 at the PLA General Hospital First Medical Center and received necessary and regular follow‐up. We developed novel models using R software to predict factors that affect survival, such as progression‐free survival and overall survival. Then, the model was optimised by evaluating the prediction efficiency in multiple dimensions. Eight hundred and forty‐nine patients with EC were included in the study. Survival‐related influences on EC patients were identified by univariate analysis and cox regression equations. In addition, a nomogram was visualised in conjunction with demographic characteristics and the above meaningful clinicopathological variables. Ultimately, through a comprehensive assessment, a random forest model (RF16) was developed for complementing the findings of the molecular classification of EC. The RF16 not only specifically characterises tumour molecules, but also enhances the generalizability of the model by replacing gene sequencing with immunohistochemistry. This study showed that the machine learning model (RF16) is low‐cost, efficient, and clinically valuable in guiding treatment for EC patients.

## 1 Introduction

Endometrial cancer (EC) is one of the most common malignant tumours of the female reproductive tract, and the incidence is on the rise due to various factors, such as population aging, socio‐cultural changes, and ecological impact. In China, EC is the second most prevalent cancer after cervical cancer, with a new incidence rate of 10.54 per 100,000 [1]. While early‐stage EC confined to the uterus has a 5‐year survival rate of 74%–91%, advanced disease with multiple distant metastases has only a 5‐year survival rate of only 20% [2]. Even with optimal treatment decisions including standardised staging surgery and adjuvant therapy, 7%–15% of EC patients with high‐risk factors still experience disease recurrence, including 50% local recurrence, 25% distant metastases, and 25% both [3]. Therefore, there is an urgent need to explore a risk factor model for EC, enabling the identification of patients at high risk of recurrence and death, and to provide them with appropriate preventive measures and prognostic management.

Although extensive researches have been devoted to the pathogenesis and progression of EC, molecular markers are being explored to help predict prognosis accurately [4, 5, 6]. Currently, reviewing a large number of clinical studies at home and abroad, predicting the prognosis of EC patients based on TCGA molecular typing and guiding clinical treatment have shown good application prospects. However, the precision medicine for patients remains uncommon due to the cost of detection and technical limitations. Compared to different gene sequencing techniques, immunohistochemistry (IHC) has been widely used in the management of EC patients due to its advantages of rapidity and simplicity [7].

Additionally, machine learning, as an intelligent tool, serves scientific research in various fields. Automated processing of large amounts of data and strong self‐learning ability are the main advantages to help construct the practical problem models. Currently, a random forest model that integrates important pathological characteristics in oncology IHC is a precision tool that can estimate prognosis by adding other clinical useful predictors [8, 9].

Therefore, this study aimed to utilise the intelligence tool and easy molecular information to construct a prognostic prediction model for EC patients, which could aid in the determination and adjustment of treatment strategies and maximise patient benefits.

## 2 Methods

### 2.1 Study Population

We, retrospectively, reviewed data of EC patients underwent hysterectomy ± adnexectomy ± lymph nodes dissection at the PLA General Hospital First Medical Center between December 2014 and June 2020. Patients eligible for inclusion in this study were those with: (a) histologically proven endometrioid adenocarcinoma and receiving initial surgical treatment; (b) complete medical records (age, body mass index [BMI], menopause state, pregnancy and gestation history, underlying comorbidities [hypertension and diabetes], endometrial hyperplasia status, tumour characteristics including tumour size, stage, histologic type and grade, lymphovascular space invasion [LVSI], lymph node metastasis, deep muscle infiltration, and immunohistochemical makers including ER, PR, P53, Ki67, and mismatch repair [MMR]); (c) no other malignancies; (d) no mental illness; and (e) regular and standardised follow‐up. Patients who did not receive the standard surgery were excluded. After these criteria were applied, a total of 849 EC patients were included in this study. Then, 80% of the included patients were randomised to the training set (*n* = 698) and 20% to the testing set (*n* = 151). To avoid overfitting, the study analysis involved internal validation cohorts, which accounted for 20%. Written informed consent was waived due to the retrospective study design. The private information from patients were anonymised and de‐identified prior to the analysis.

### 2.2 Treatment and Follow‐Up

Patients received at least a total hysterectomy. According to the National Comprehensive Cancer Network guidelines, appropriate adjustments to the scope of surgery, including lymph node dissection or no dissection, are allowed [10]. Patients at different risk levels received regular follow‐up: every 3–6 months for the first 3 years, then every 6 months or annually until 5 years. Each visit included a physical and gynaecological examination and necessary ancillary tests, such as CA125 test, vaginal stump cytology, and imaging [11, 12]. Follow‐up visits began on the day of surgery and continued until October 2021.

### 2.3 Immunohistochemistry

All post‐operative specimens from each patient were processed at the pathology department with the same criteria. Selected wax blocks were cut into consecutive 4‐μm‐thick white sections and subjected to immunohistochemical detection using the EnVision method. Evaluation of molecular staining was performed by two senior pathologists independently under double‐blind conditions. The results were shown as the percentage of positive tumour cells. When the results of the two senior pathologists differed by ≤ 10%, they were considered concordant. However, if the difference was > 10%, it was re‐evaluated by a non‐blinded method until agreement was reached. Finally, the average of the percentages assessed by the two senior pathologists was determined as the final result. Information on the antibodies involved in the study is given in the following table.

**Table:** 

Antibody	Specification	Company
Anti‐oestrogen receptor alpha	ab32063/100ul	Abcam
Anti‐progesterone receptor	ab16661/100ul	Abcam
Anti‐MLH1	ab92312/100ul	Abcam
Anti‐MSH2	ab227941/100ul	Abcam
Anti‐MSH6	ab92471/100ul	Abcam
Anti‐PMS2	ab110638/100ul	Abcam
P53	ab32049/100ul	Abcam
Ki67	ab92742/100ul	Abcam

### 2.4 Definition

The tumours were histologically classified as endometrioid adenocarcinoma according to the World Health Organisation classification of tumours [13]. Clinicopathological characteristics were included in the analysis of the results.

#### 2.4.1 Molecular Markers by IHC Definition

The ER/PR (+) was defined as > 5% of tumour cells with brownish‐yellow granules in the nucleus [14]. A deficient mismatch repair [dMMR] means that any of the four proteins MLH1, MSH2, MSH6, and PMS2 are expressed deficiently, while a proficient mismatch repair [pMMR] means that these four proteins are fully expressed [15]. When > 40% of tumour cells showed Ki‐67 (+), the sample would be defined as high Ki‐67 expression [16]. Both complete lack of expression and overexpression (> 70%) were deemed as P53 abnormal or mutated, while the wild‐type pattern was considered P53 normal [17].

#### 2.4.2 Survival Definition

Overall survival (OS) is defined as the period from the date of surgery to the time of death. Progression‐free survival (PFS) is defined as the period from the end of initial treatment to the first occurrence of disease progression or death from any cause.

### 2.5 Statistical Analysis

Baseline characteristics were summarised as median (range) or average ± standard deviation (*X* ± SD) of numerical variables, frequency, and proportion of categorical variables. Due to the competing risks in this study, univariate and multivariate analyses were performed by *p* value and hazard risk (HR). Cox proportional risk models and random forest plots were used for multifactor analysis. The accuracy of machine learning model was verified using the area under the curve (AUC) and the *C*‐index. An AUC of 1.0 indicated a perfect concordance, whereas an AUC of 0.5 indicated no relationship.

All statistical analyses were performed using SAS version 9.1 (SAS Institute, Cary, North Carolina, USA); SPSS version 23 (SPSS; IBM Corp, Armonk, New York, USA); and R software version 4.2.1 (http://www.r‐project.org) with the ‘rms’, ‘survival’, ‘survminer’, ‘pROC’, ‘precrec’, ‘pec’, ‘survcomp’, ‘ggplot2’ packages and ‘Python3 (3.9.9): scikit‐learn (0.23. 1)’ in random forest. Statistical analyses involved the training and validation cohorts. Two‐sided *p* values < 0.05 were considered statistically significant.

## 3 Results

### 3.1 Patients and Tumour Characteristics

A total of 849 EC patients were included in the study, with 698 in the training cohort and 151 in the testing cohort. The median age of all patients at the time of surgery was 58 years (range: 26–84 years). The average BMI of enrolled patients was 26.40 ± 4.20 kg/m^2^ (median: 25.80 kg/m^2^, range: 17.60–39.40 kg/m^2^). Demographic data and clinicopathological parameters of patients in two cohorts are provided in Table 1.

**TABLE 1:** Demographic and clinicopathological characteristics.

	Training cohort (*n* = 698)	Testing cohort (*n* = 151)
Age (years)		
< 50	188 (0.27)	24 (0. 16)
50–70	468 (0.67)	108 (0.72)
≥ 70	42 (0.06)	19 (0. 12)
BMI (kg/m^2^)		
< 25	274 (0.39)	61 (0.40)
25–30	299 (0.43)	72 (0.48)
≥ 30	125 (0. 18)	18 (0. 12)
Stage		
I	532 (0.76)	81 (0.54)
II	61 (0.09)	19 (0. 12)
III	84 (0. 12)	33 (0.22)
IV	21 (0.03)	18 (0. 12)
Menopause		
Yes	459 (0.66)	115 (0.76)
No	239 (0.34)	36 (0.24)
Pregnancy_history		
Yes	661 (0.95)	141 (0.93)
No	37 (0.05)	10 (0.07)
Gestation		
0	54 (0.08)	12 (0.08)
1	333 (0.48)	71 (0.47)
2	213 (0.31)	46 (0.30)
3	74 (0. 11)	19 (0. 13)
4	19 (0.03)	1 (0.01)
5	3 (0.004)	0 (0.00)
6	2 (0.003)	2 (0.01)
Hypertension		
Yes	252 (0.36)	45 (0.30)
No	446 (0.64)	106 (0.70)
Diabetes		
Yes	137 (0.20)	32 (0.21)
No	560 (0.80)	119 (0.79)
Endometrial_hyperplasia		
Atypia	251 (0.36)	14 (0.09)
Without_atypia	12 (0.02)	21 (0. 14)
None	435 (0.62)	116 (0.77)
Histologic_differentiation		
High	330 (0.47)	86 (0.57)
Low	368 (0.53)	65 (0.43)
Deep_myometrial_invasion		
Yes	164 (0.23)	71 (0.47)
No	534 (0.77)	80 (0.53)
LVSI		
Yes	77 (0. 11)	65 (0.43)
No	621 (0.89)	86 (0.57)
Tumour_size		
< 2 cm	216 (0.31)	102 (0.68)
≥ 2 cm	482 (0.69)	49 (0.32)
Lymph_node_metastasis		
Yes	59 (0.08)	46 (0.30)
No	536 (0.77)	105 (0.70)
P53		
Mute	147 (0.21)	36 (0.24)
Wild	548 (0.79)	115 (0.76)
MMR		
Deficient	131 (0. 19)	43 (0.28)
Proficient	533 (0.76)	108 (0.72)
ER		
Positive	639 (0.92)	126 (0.83)
Negative	58 (0.08)	25 (0. 17)
PR		
Positive	633 (0.91)	114 (0.75)
Negative	64 (0.09)	37 (0.25)
Ki‐67		
Over‐expression	377 (0.54)	94 (0.62)
Normal	316 (0.45)	57 (0.38)
Surgical_therapy		
Adnexectomy	103 (0. 15)	31 (0.21)
Adnexectomy + Lymphadenectomy	595 (0.85)	120 (0.79)
Adjuvant_therapy		
Progesterone	296 (0.42)	8 (0.05)
Chemoradiotherapy	173 (0.25)	93 (0.62)
None	24 (0.03)	50 (0.33)

### 3.2 Selecting Factors Associated With Survival Prognosis

In the training cohort, 33 (4.73%) patients relapsed and 18 (2.58%) patients died. In the testing cohort, a total of 32 patients with advanced EC relapsed and 20 patients died. The 5‐year OS and PFS for all patients were 93.75% and 95.83%, respectively.

**FIGURE 1:**
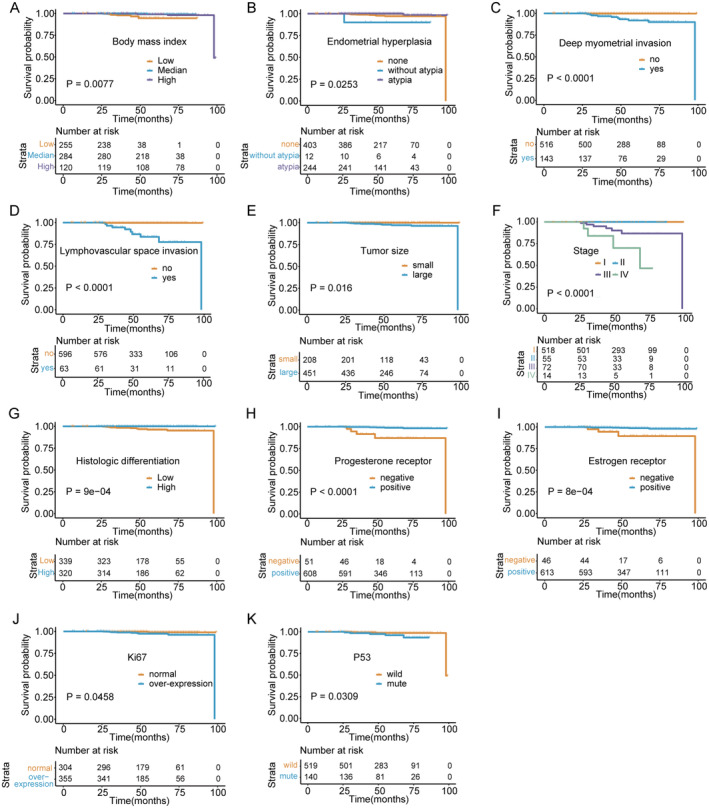
Univariate analysis for OS. (A) The effect of BMI on overall survival was analysed in three groups of patients with endometrioid adenocarcinoma (low = BMI < 25 kg/m^2^, median = between 25 and 30 kg/m^2^, high = BMI ≥ 30 kg/m^2^). (B) Impact of history of endometrial hyperplasia, including atypical and other benign hyperplasia, on overall survival. None means unknown. (C) Effect of deep myometrial invasion or not on overall survival. (D) Effect of Lymphatic vascular interstitial infiltration (LVSI) or not on overall survival. (E) Impact of tumour differentiation on survival outcome, with high representing highly differentiated and low representing moderately or poorly differentiated. (F) Impact of tumour 2009FIGO staging on survival outcomes. (G) The effect of tumour size on survival time, small means < 2 cm, large means ≥ 2 cm. (H–K) Molecular expression status involving ER, PR, Ki67, and P53 for survival effect.

The coxph function was used for univariate analysis to investigate the impact of various factors on prognosis. The study confirmed that clinical characteristics, such as BMI (*p* = 0.008) and history of endometrial hyperplasia (*p* = 0.025) in patients with endometrioid adenocarcinoma significantly affect survival outcomes (Figure 1A,B). Deep myometrial invasion acted as a risk factor for endometrial carcinoma, increasing mortality (HR = 31.3, [95% CI: 7.9–124.1], *p* < 0.001) (Figure 1C). Patients with pathological features of LVSI had a 30‐fold risk of death than patients without LVSI (HR = 30.6 [95% CI: 5.2–178.8], *p* < 0.001) (Figure 1D). In addition, tumour size (*p* = 0.016), stage (*p* < 0.001), and differentiation (*p* < 0.001) were significantly associated with OS (Figure 1E–G). Immunohistochemical results showed 90% reduction in risk of death in PR‐positive patients (HR = 0.1 [95% CI: 0–0.9], *p* < 0.001) and 80% reduction in risk of death in ER‐positive patients (HR = 0.2, *p* < 0.001) (Figure 1H,I).

**FIGURE 2:**
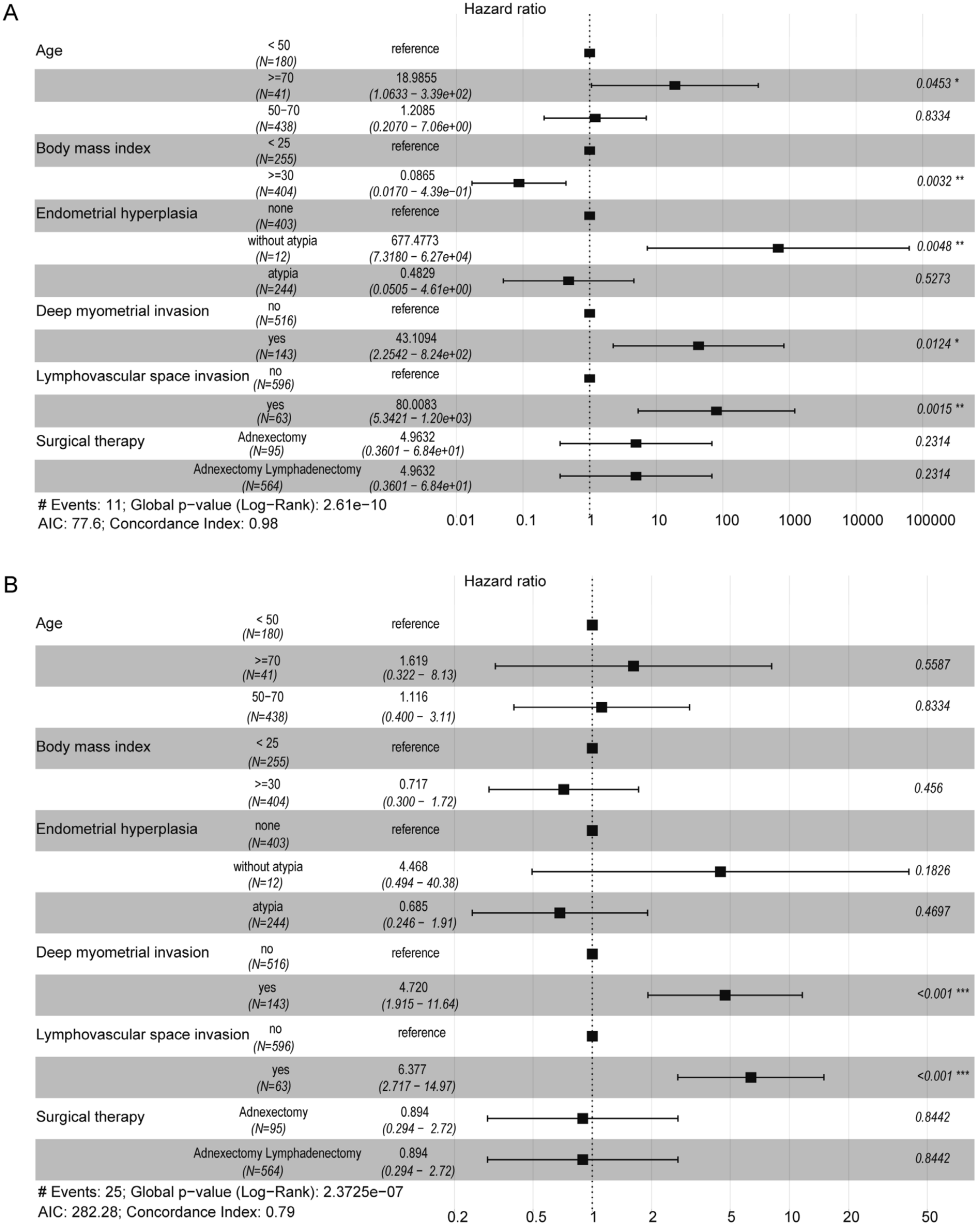
Forest for OS and PFS. (A) Multifactorial analysis of overall survival in patients with endometrioid adenocarcinoma. (B) Multifactorial analysis of progression‐free survival in patients with endometrioid adenocarcinoma.

**FIGURE 3:**
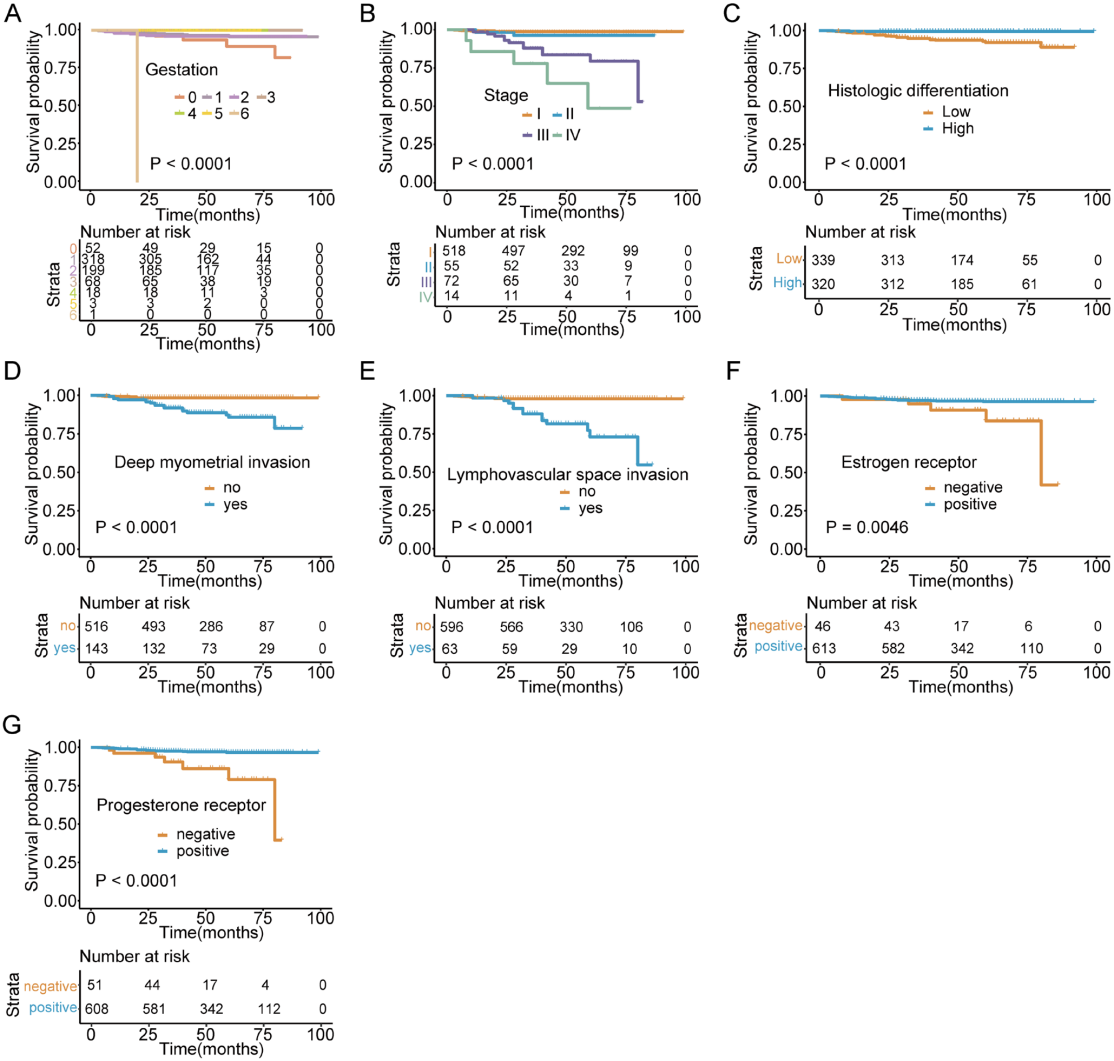
Univariate analysis for PFS. (A–G) The effect of gestation, tumour stage, histological differentiation, deep myometrial invasion, LVSI, and ER and PR expression status on progression‐free survival.

However, patients with both high Ki‐67 expression (HR = 4.2 [95% CI: 1.3–13.7], *p* = 0.046) and P53 mutations (HR = 3.4, *p* = 0.031) were at increased risk of death (Figure 1J,K). Besides, BMI (*p* = 0.003), atypical hyperplasia (*p* = 0.005), deep myometrial invasion (*p* = 0.012), and LVSI (*p* = 0.002) were considered as independent hazard factors for OS in multivariate analysis (Figure 2A). Univariate analysis of PFS in patients with endometrioid adenocarcinoma demonstrated that gestation (*p* < 0.001) and tumour stage (*p* < 0.001) were meaningful predictors of recurrence (Figure 3A,B). The results illustrated that patients with highly differentiated tumour histology had a 90% reduced risk of disease recurrence and progression (HR = 0.1 [95% CI: 0–0.2], *p* < 0.001) (Figure 3C). Consistent with the OS results, both LVSI (HR = 10.8 [95% CI: 2.8–42.4], *p* < 0.001) and deep myometrial invasion (HR = 7.9 [95% CI: 3–20.7], *p* < 0.001) were significant poor factors in the evaluation of recurrence (Figure 3D,E). Also, ER (*p* = 0.005) and PR (*p* < 0.001) positive staining were protective factors for PFS (Figure 3F,G). Additionally, the results of multivariate analysis manifested that deep myometrial invasion (*p* < 0.001) and LVSI (*p* < 0.001) were independent risk indicators correlated with PFS (Figure 2B). Besides, the prognostic efficacy of multiple combinations of conditional factors was shown (Data S1).

### 3.3 Nomogram Construction for Prognostic Prediction

**FIGURE 4:**
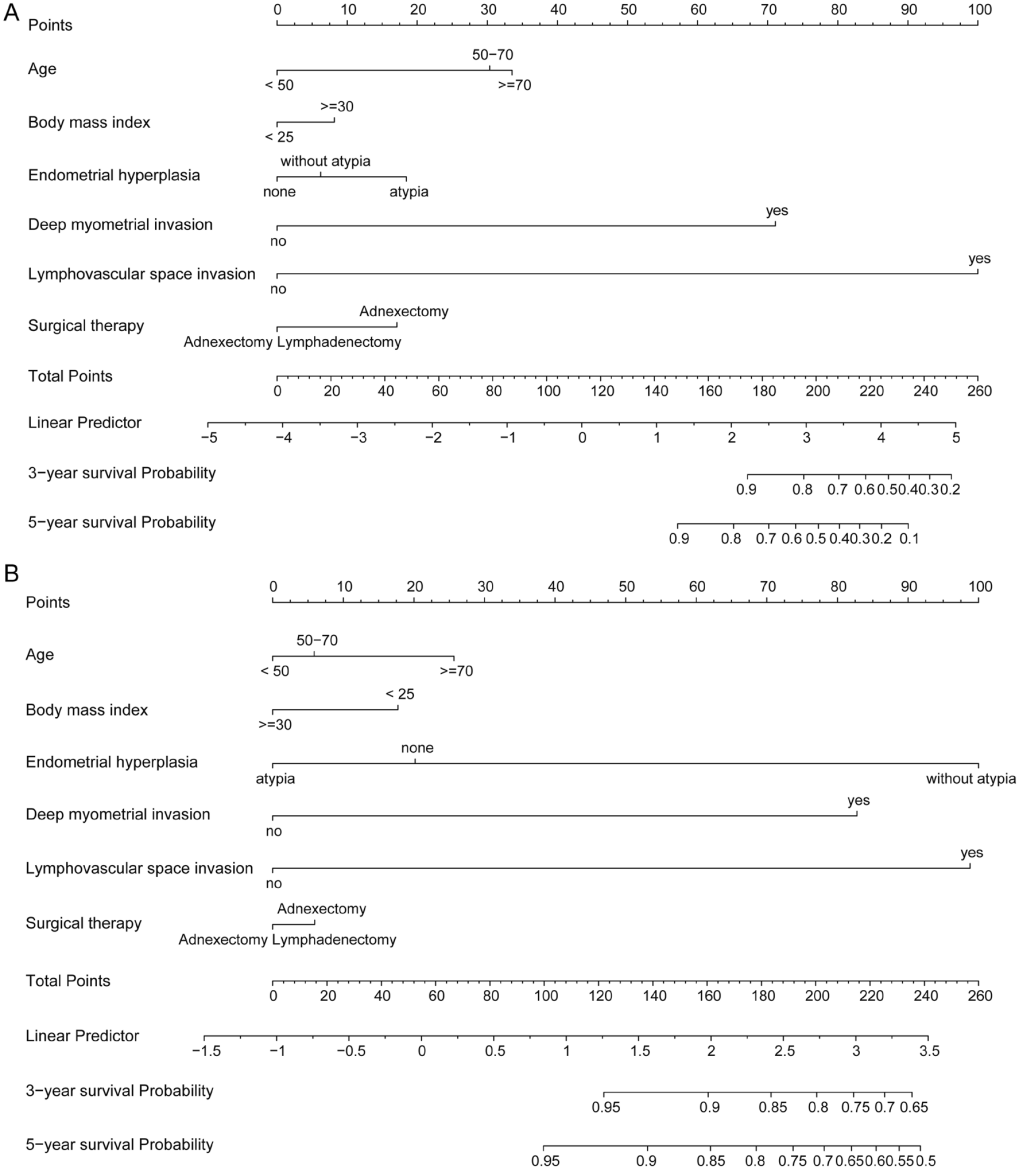
Nomogram for OS (A) and PFS (B).

The training set is basically consistent with the baseline of the testing set, as demonstrated in Table 1. A nomogram was constructed by integrating demographical and clinical variables from the multivariate model mentioned above for OS and PFS, respectively (Figure 4A,B). As an intuitive form of the regression equation, the nomogram was used to calculate the total score and eventually transformed into a survival probability through the cumulative integration of each independent variable.

### 3.4 Efficiency of the Random Forest Model for Prognosis Prediction

**FIGURE 5:**
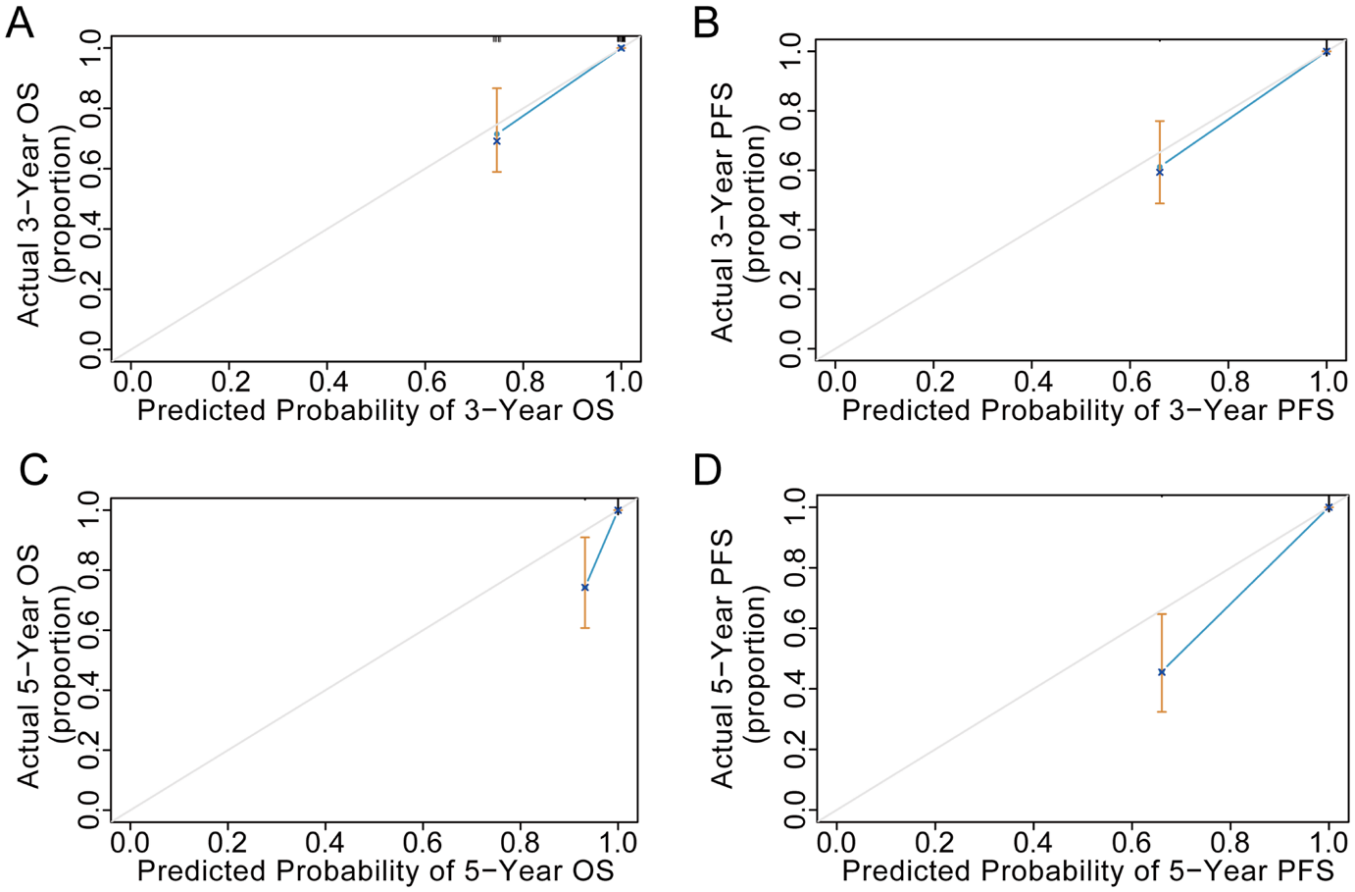
Calibration curves for prediction models in the testing cohort. (A, B) Calibration curves for 3‐year OS and PFS. (C, D) Calibration curves for 5‐year OS and PFS.

**FIGURE 6:**
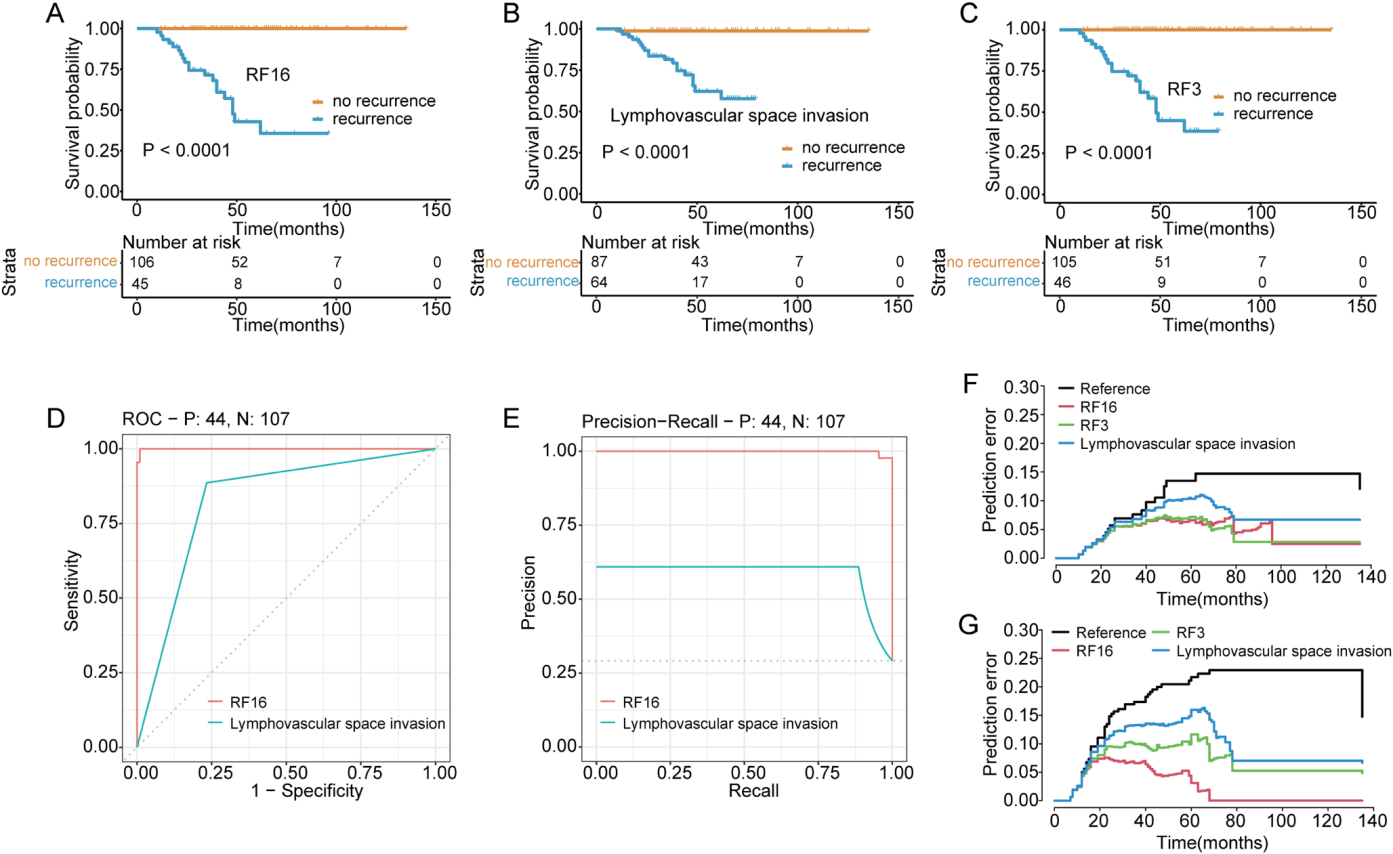
Comparison of efficiency and prediction error between RF16 and LVSI. (A) Testing the RF16 model to predict survival outcome. (B) LVSI one‐way analysis for predicting prognostic outcome. (C) Testing the RF3 model to predict survival outcome. (D) ROC curves of the RF16 model and LVSI single factor. (E) Precision‐recall curves of the RF16 model and LVSI single factor. (F, G) Cumulative OS and PFS prediction error curves over time in the testing set.

**FIGURE 7:**
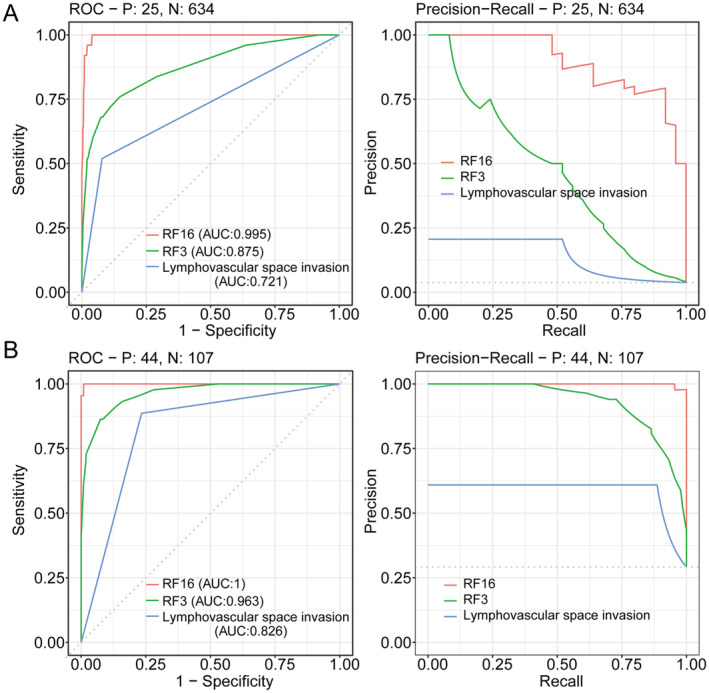
Comparison of efficiency and prediction error among RF16, RF3, and LVSI in the training cohort (A) and in the testing cohort (B).

A random forest model (RF16) was constructed combining age, BMI, gestation, menopause, endometrial hyperplasia, stage, tumour size, histological differentiation, LVSI, deep myometrial invasion, surgical therapy, P53, Ki67, MMR, ER, and PR to complement the findings on molecular classification of EC. Through machine learning methods, available molecular markers can be combined with other meaningful traditional variables. For the prediction of survival outcomes, RF16 showed good discrimination accuracy with an AUC of 0.991 and a *C*‐index of 0.938 (95% CI: 0.868–0.982) in the testing cohort. The calibration curves reflected the consistency and correlation between the actual and expected outcomes in the testing cohort (Figure 5). The assessment of prognosis was developed among RF3, RF16, and LVSI by sensitivity analysis (Figure 6). Additionally, RF16 model showed a higher accuracy and lower likelihood of error occurrence than RF3 combining two clinical factors (age and BMI) and LVSI (Figure 7).

## 4 Discussion

EC continues to be a significant burden for families and gynaecologic oncologists, with increasing incidence in the country [18]. Due to the growing amount of published data suggesting that EC is a heterogeneous entity with different treatment options and post‐treatment follow‐up, most guidelines recommend complete surgical staging and patient‐oriented prognostic management based on risk factors [19, 20, 21]. In addition, molecular characterisation of EC has been incorporated into the 2023 FIGO staging criteria, and molecular typing guides prognostic stratification and risk management. Therefore, it is timely to emphasise molecular staging of EC. According to advances in tumour biology, cancer cells located in border or core regions, they exhibit different phenotypic states and different microenvironmental characteristics. Previous low‐throughput studies based on IHC relied heavily on the selection of regions of interest and the design of protein panels. In contrast, spatial multi‐omics techniques allow unbiased discovery of the expression of key genes that control the fate of tumourigenesis. Therefore, based on the advantages of newer assay technologies, the incorporation of spatial transcriptomics into prognostic models can effectively reduce bias and increase the clinical translational value of models.

Risk factors affecting the prognosis of EC have been extensively studied in recent years. However, the association of poor survival prognosis with rates of guideline‐concordant treatment, treatment timing, demographic characteristics, biomarker expression, and care disparities remains unclear [22]. Although individual pathological differences and medical inequalities contribute to survival differentiation for EC patients, no single factor is solely responsible for the outcome disparity among the patients [23].

Therefore, a model that used cost‐effective detection and analysis technology to establish links between various prognostic factors is needed today. Using IHC instead of expensive genetic testing to reveal complicated tumour microenvironment and partial pathological features can maximise the benefit of more patients [24, 25, 26, 27, 28, 29].

In this study, we developed a machine learning model in which the measures were simplicity, feasibility and efficiency to predict the outcomes of EC patients. The following 16 independent variables identified by machine learning methods were used for new prognosis prediction: age, BMI, gestation, menopause, endometrial hyperplasia history, stage, tumour size, histological differentiation, deep myometrial invasion, LVSI, surgical therapy, the expression of P53, Ki67, MMR, ER, and PR.

To better discern disease progression and potential prognostic trends, mutations in the molecular features and genetic information of the disease were emphasised in the model. Previous studies have indicated that microsatellite instabilities are associated with high levels of tumour‐infiltrating lymphocytes and antigen loads [30, 31, 32], which can improve the detection rate of Lynch syndrome and also serve as a marker for screening immune‐beneficiary populations. Besides, ER/PR was the most commonly expressed as an indication for hormonal therapy. In addition, the least desirable P53 mutation status was considered in combination with targeted therapy to strive for the possible benefit. The machine learning model achieved excellent predictive power using only routine data at the time of post‐operative decision‐making.

To our knowledge, this is the first real‐world research on prognosis prediction model that combines endometrial carcinoma immunohistochemical molecular characteristics and complete clinicopathological data, and has high sensitivity and specificity, along with popular clinical application value. Based on the result, more genetic testing costs can be avoided, while a large number of molecular research results can be used to achieve precise treatment. To address bias, the rigorous technique of random forest imputation has also been applied in this model.

An accurate prognosis is of utmost importance in avoiding damage to patients' interests, including side effects of over‐medication, frailty assessment in elderly patients, personal economic losses, and the waste of social medical resources. This excellent machine learning model proves that artificial intelligence systems serve human beings effectively. Machine learning models have the potential to improve the efficiency of medical staff, integrate patient disease characteristics quickly, and guide the development of scientifically sound remedies. Besides, the machine learning approach is also helpful for establishing individualised electronic files for tumour patients, storing specific and large numbers of clinical and pathological indicators, promoting full, standardised, and fine disease control throughout the life cycle, and easily realising precise health management.

Although machine learning demonstrated better performance compared to conventional approaches, a few limitations should be noted in this study. The limitations of single‐centre retrospective studies have to be acknowledged. Despite expanding our sample size and validating our conclusions with a test set, there may still be selection bias affecting extrapolation of conclusions. Additionally, the accuracy of machine learning approaches relies on data accuracy strictly, and molecular expression classification based on thresholds can be somewhat subjective. In addition, some parameters were not included in the analysis, such as ethnic group, blood type, and POLE mutation. However, the highlight of this study was not to develop a prediction model using a broad set of predictors, but to harness a limited set of clinical data that is cost‐effective and currently available in the treatment unit.

Future research can focus on exploring the wider range of applications for machine learning. For instance, machine learning has the potential to provide specific treatment details, such as drug dosage and frequency, as well as intervention indication, making it a popular and useful tool in the future.

## Author Contributions

**Di Wu:** conceptualization (lead), data curation (equal), resources (lead). **Zhifeng Yan:** conceptualization (equal), visualization (equal). **Mingxia Li:** data curation (equal), visualization (equal). **Mingyang Wang:** methodology (equal), software (equal). **Yuanguang Meng:** project administration (equal), supervision (equal).

## Conflicts of Interest

The authors declare no conflicts of interest.

## Supporting information


**Data S1** This table presents the statistical results of the impact on survival of patients with endometrial cancer, and we consider any combination of conditions with a *p*‐value of < 0.05 to be a statistically significant impact factor. The first column is the influencing factor, the second column is the statistical *p*‐value, and the third column is the hazard ratio.

## Data Availability

The PLA library.
